# Emotion Induced Monoamine Neuromodulator Release Affects Functional Neurological Disorders

**DOI:** 10.3389/fcell.2021.633048

**Published:** 2021-02-15

**Authors:** Fei Liang, Qiuyue Xu, Mingchen Jiang, Rou Feng, Shan Jiang, Bin Yuan, Shijun Xu, Ting Wu, Fushun Wang, Jason H. Huang

**Affiliations:** ^1^Institute of Brain and Psychological Science, Sichuan Normal University, Chengdu, China; ^2^School of Medicine, Nanjing University of Chinese Medicine, Nanjing, China; ^3^Jiangsu Key Laboratory of Pediatric Respiratory Disease, Affiliated Hospital of Nanjing University of Chinese Medicine, Nanjing, China; ^4^School of Pharmacy, Chengdu University of Traditional Chinese Medicine, Chengdu, China; ^5^Department of Neurology, The First Affiliated Hospital of Nanjing Medical University, Nanjing, China; ^6^Department of Neurosurgery, Baylor Scott & White Health, Temple, TX, United States; ^7^Department of Surgery, College of Medicine, Texas A&M University, Temple, TX, United States

**Keywords:** functional neurological disorders, three primary emotions, monoamine neuromodulators, emotional, conversion disorder, prediction error

## Abstract

Functional neurologic disorders (FNDs), also called conversion disorder (previously called hysteria), can show almost all the symptoms of other neurological diseases, including both physical (for example, seizure, weakness, fatigue) and psychological (for instance, depression, anxiety) symptoms. In spite of our general knowledge about emotional processes and developmental defects in the formation of these somatic symptoms, there is still no systemic and comprehensive research on the effects of emotional developmental variables in FND. Recently, both experimental and theoretical emotion studies have been greatly increased, such as prediction error, conceptual act model, basic emotional theory, and monoamine neuromodulator based three primary emotions. In addition, a large amount of evidence has confirmed the role of psychosocial adversity (such as stressful life events, interpersonal difficulties) as an important risk factor for FND. Here, we review recent advances about emotional stress on FND, and pay special attention to the effects of monoamine neuromodulators, such as how norepinephrine and serotonin affect behaviors. Then, we discuss the significance of these changes for FND, which may contribute to clarifying the pathogenesis of FND, and thus provide potential therapeutic drug targets or psychological intervention methods in the future.

## Introduction

Functional neurologic disorders (FNDs) are common sources of disability and the second most common referral to neurological outpatients (Ludwig et al., 2018). FND covers a variety of neurological symptoms, which are similar to almost any neurological illness. Symptoms are diverse and can include weakness, movement disorders (tremor, jerks, and dystonia), sensory symptoms, cognitive deficits, and seizure-like events (commonly known as dissociative seizures or non-epileptic seizures). Fatigue and persistent pain are also commonly experienced as part of the disorder. Symptoms can present acutely and resolve quickly or can be long lasting. Even though they are very real neurological symptoms, they are not real neurological diseases, thus FND patients are often being misdiagnosed, leading to improper treatment. The name FNDs was used by DSM-5 (Diagnostic and Statistical Manual of Mental Disorders, fifth edition) to replace the term “conversion disorder” and removed “psychogenic,” and the criterion of psychological stress as a perquisite in DSM-4. FND is defined in DSM-5 as the presence of one or more symptoms of altered voluntary or sensory function, with clinical findings providing evidence of incompatibility between the symptom and recognized neurological or medical conditions (Voon et al., 2016).

Although reference to the psychological origin of FND has been removed from the criteria list in DSM-5, many theories still regard FND as physical disturbances that generally occur along with malicious thoughts, emotions, or health-related concerns (Bègue et al., 2019). Or FND is still considered to be derived from adverse events induced stressful emotional disorders (Stone, 2016; Fend et al., 2020). Stressful experience is the main risk factor in most cases of FND (Christensen et al., 2020), and is related to the mechanism and treatment of FND (Ludwig et al., 2018). Moreover, more evidence points to the causal role played by early life stress in the development of FNDs (Keynejad et al., 2019). The putative Bio-psycho-social mechanisms underlying FND are complex and have been extensively reviewed (Voon et al., 2016), but it is still unclear about how emotional stress affects neurological dysfunction and related neuromodulation conditions? The research in this field may affect our understanding of the pathogenesis of FND, and has broader significance for our understanding of functional diseases that affect other body systems. This review provides a critical review of the literature on the stressful life events in FNDs, and we propose that stress and stress-induced changes in neuromodulators contribute the FNDs.

## Epidemiology of FND

Functional neurologic disorder is defined by neurological symptoms that encompasse functional movement disorders, functional weakness, functional numbness, psychogenic non-epileptic seizures etc. FND accounts for approximately 6% of neurological outpatients and the presumptive community morbidity at a rate of 4–12 per 100,000 per year. About 10% of cases are combined with neurological diseases. This disease is more common in women, especially those between the ages of 35 and 50 (Carson and Lehn, 2016), which might be due to several factors, such as sexual hormone release to facilitate some mental disorders, or women are easy to get sexual harassment. One study reported a preliminary diagnosis of 3781 patients in the UK Neurology Center, of which FND accounted for 16%. It is worth noting that only 5.4% of these patients were initially diagnosed as FND. About 30% of symptoms are only described to some degree or not explained by the disease at all (Stone et al., 2010). Another study shows that American soldiers are at growing risk of FND. The incidence rate was estimated to be 29.5 per 100,000 persons between 2000 and 2018 as a whole (Garrett et al., 2020). Thus, FND is very common, occurring in up to one-third of patients in neurological outpatient clinics (Voon et al., 2016), and needs reliable diagnostic information to allow neurologists to accurately diagnose them, which can greatly reduce the pain caused by FND and ultimately improve results. However, limited understanding on the pathophysiology has impeded the correct diagnosis and therapies of FND.

**FIGURE 1:**
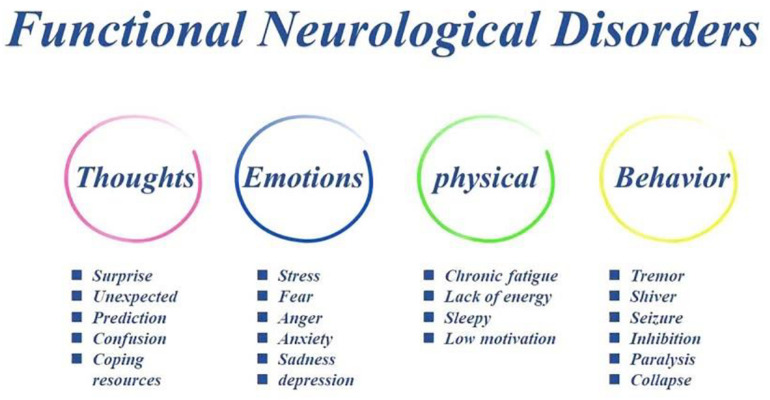
The symptoms of FND patients. Traumatic experiences in FND patients can induce problems in thoughts (such as expected prediction and learned helplessness), which in turn induce some stressful emotions (anxiety and depression), thus bad interceptive sensation and improper behaviors (tremor and shiver, paralysis).

The importance of psychosocial adversity in FND cannot be underestimated given that rates of early life and proximal adverse events have been repeatedly found to be higher in FND samples, and stressful life-events including abuse/neglect, ongoing relationship disturbances, occupational stress, and caring responsibilities are commonly reported (Pick et al., 2019). FND is defined by symptoms which have been labeled as “psychogenic,” “hysterical,” “non-organic,” or “medically unexplained” and symptoms cannot explained by identifiable neurological pathology. These symptoms include negative emotions (anxiety and depression) and interceptive sensation (stomach ache), negative cognition (such as improper thoughts, prediction error), or behavioral changes (tremor and shiver, paralysis) (Figure 1). The symptoms were originally formulated as hysterical conversion by Breuer and Freud (1895/1995). And following psychodynamic theories proposed that emotions play a crucial role in the etiology of FND. However, some investigators recently questioned the importance of emotions in the etiology of the disorder, because emotional dysregulation is not always apparent in FND (Espay et al., 2018a), and contemporary explanations have moved away from psychodynamic trauma-focused models and instead emphasize dysfunction of higher-order cognitive processes (Voon et al., 2010b).

Several etiological models have been proposed in the last century. Bio-psycho-social medical model frameworks acknowledge a variety of predisposing (psychosocial adversity, gender etc.), precipitating (mental health problems, personal conflict), and perpetuating (illness belief/expectation) factors that can contribute to FND (Pick et al., 2019). However, the exact mechanism underlying FND symptoms are still not fully understood, and there is no well-accepted explanatory model. Recent studies still found that a history of maltreatment and stressful life events is common in FND patients (Ludwig et al., 2018). The traumatic experiences of FND might be attributed to bad thoughts (such as expected prediction and learned helplessness), which lead some negative emotions (anxiety and depression) and improper interceptive sensation, thus behavior dysfunctions (tremor and shiver, paralysis) (Mesurado et al., 2018; Figure 1).

## Emotional Processing in FND

### Freud’s Conversion

Functional neurologic disorder symptoms are varied and include abnormal control of movement, episodes of altered awareness resembling epileptic seizures and abnormal sensation and are often comorbid with chronic pain, fatigue, and cognitive symptoms. Many studies suggest that FND is emotional expressions of distress (Sojka et al., 2018). In their seminal work, Breuer and Freud (1895/1995) suggest that FNDs are due to “the excitation arising from the affective idea is converted into a somatic phenomenon.” Freudian theory of conversion suggested that the major symptoms of FND are due to internal conflict. Later on, many studies confirmed that FND patients have abnormal emotions and physiological changes, such as increased salivary cortisol, increased heart rate. Consistently, experimental studies found that FND patients’ emotional expression and skin conductance have changed (Pick et al., 2016). In addition, FND patients showed altered somatosensory responses at stresses. However, this psychogenic model of FND has moved from psychodynamic conversion model to a multi-network model, involving abnormalities within and across brain circuits implicated in self-agency, emotion processing, attention, homeostatic balance, interoception, multimodal integration, and cognitive/motor control among other functions (Spence et al., 2000; Seeley, 2019; Drane et al., 2020).

### Prediction Error

**FIGURE 2:**
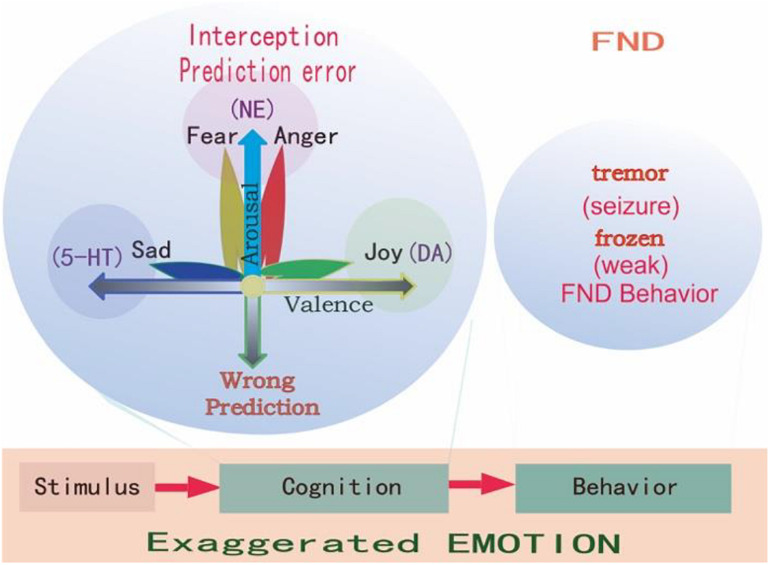
Prediction errors in FND. Emotions are evoked by stimulations, and all stimulations have two features: whether it happens as predicted (arousal), and whether it fits into our physiological needs (valence). So it is said “All emotions are induced as feelings of hedonic pleasure and displeasure (valence, horizontal dimension), with some degree of arousal (prediction error, vertical dimension) (Russell and Barrett, 1999).” The FND patients have experienced stressful childhood, and so they have wrong coping styles at stressful situations, and have an exaggerated interception and emotions and behaviors, such as frozen or tremor, which are very common in FND patients.

Even though the social-psycho-biomedical perspective highlights psychological abnormalities and emotional problems, the psychosocial etiology for FND is still unclear. A recently developed Bayesian predictive coding framework offers an explanation for FND (Edwards et al., 2012; Sojka et al., 2018). Edwards et al. (2012) described an aberrant “prior” expectation in FND patients, and proposed a predictive coding model of impairment in expectation. The predictive coding model suggested that the brain used knowledge about the world to predict about the sensations (Friston, 2010). The predictions first depend on previous experiences, which might be different from current sensations, and constitute the prediction error (Barrett and Simmons, 2015; Cowen and Keltner, 2018). The functions of emotion and emotional induced behaviors work to minimize the difference, to change the unexpectancy, or to keep the homeostasis (Figure 2). For example, you are driving a car with your expectancy that the other cars will drive at the control speed, but suddenly a car speed very fast and cut in front of your car. You are scared for the unexpectancy, and you will be angry and try to fight with the driver of the car. This is normal emotional reaction, but if it happens for a baby, who try to show his internal states, but the caregiver failed to act properly, the child will use another way to show his internal states, such as altered interoceptive sensations (e.g., stomach ache), together with exaggerated unconscious emotional expression or behavioral responses, which might develop into coping style and somatic symptoms in FND adults (Sojka et al., 2018).

The predictor error theory suggested that the brain perceives the sensor input with past sensorial experiences, which is also called “predictive coding,” and then categorizes the sensations to evoke a specific action plan; the error between the prediction and the actual input is propagated and used as a feedback to update the internal model (Pacella et al., 2017). Contemporary motor theory proposes that motor control follows a feed-forward model in which self-generated movements are accompanied by a sensory prediction of the motor outcome. The movement prediction usually matches the sensory outcome giving rise to a sense of self-agency. A mismatch may thus give rise to the sensation that we are not in control of our movements (Voon et al., 2010a). Kranick et al. further assessed an implicit measure of agency during voluntary movements in FND patients using an action-binding task. Participants viewed a rotating clock and made judgments of when actions and outcomes occurred in three conditions: an action alone (button press), an outcome alone (tone), or an action-outcome pairing (button press-tone). This action-outcome binding is believed to underlie our subjective sensation that our actions are associated with an effect. Kranick et al. showed that patients with FND had a decrease in action-outcome binding, consistent with their decreased sense of agency.

Consistently, Schultz et al. (1997) predicted that the major function of dopamine (DA) is prediction error. It is a very interesting study, which used conditioning experiments. When the animals receive a small quantity of apple juice, the DA neurons fire action potentials, which means DA is a signal of reward. If light is turned on every time the apple juice is given, DA neurons will fire at the light, which means the stimulus-reward associated is learned, and DA learns to predict the apple juice. However, once the association between the light and the apple juice is fully learned to be a kind of prediction, and DA neurons stopped firing. It seems that the DA neuron is kind of teaching to remove the “error” between the predicted light and the reward. So Schultz proposed that DA predicts the un-expectations about the sensation with the predicted reward (Schultz et al., 1997).

### Conceptual Act Model

Similar to the prediction error, the constructionist model, which is also called the “conceptual act model,” suggests that we all predict about the new sensation with knowledge of prior experiences (Lindquist and Barrett, 2012). For example, only if we have the experience of having seen a fly on a stool, we have the disgust emotions when we see the fly sit on a bread. So Barrett et al. (2007) suggested that emotions are “situated conceptualizations,” because the new sensation is tailored to the immediate environment with previous experiences (Barrett et al., 2007).

“Conceptual act” is a kind of unconscious process that automatically and effortlessly using previous knowledge to predict current sensations. However, if the immediate sensation is different from the prediction, or if there is the prediction error, some forms of interception sense from the body, such as somatic, visceral, can be evoked. These interceptive senses are also called “core affects,” which means the bodily changes (physiological changes) (Figure 2). “Core affects can be realized by visceral control systems to help the organisms deal with motivationally salient stimuli” (Posner et al., 2005). “Core affect is a homeostatic barometer to sense the internal states usually in an external surrounding” (Russell, 2003). People automatically make meaning of their core affects, such as dizzy or nausea, and use these meaning to conceptualize the outside world, such as a fly. Or people can make the physical symptom, such as tiredness as feeling of boring (Lindquist and Barrett, 2012). For FND patients, these conceptualizations might be exaggerated due to evil experiences in childhood.

### Appraisal Theory of Emotion

Similar to the predictive coding model, the appraisal theory also proposes that emotions are innate states which are activated by stimulation events. In many appraisal models, the assumption is that the brain contains a series of specific cognitive appraisal mechanisms. Ekman (1992) said all emotions differ in the stimulation events, appraisals, behavioral response, and physiological responses. Lazarus (1999) said emotion is not psychologically meaningful unless it is related to an object, emotion is perceived as part of an object itself rather than one’s reaction to it. Every object has two features: whether it is fit for our physiological needs (hedonic value), and whether it happens as expected (arousal) (Gu et al., 2016; Syliadis et al., 2018). These two features correspond to the two core affects: the hedonic value that represents physiological needs and the safety value represents the way the stimulus happens (Gu et al., 2019a). The two-dimensional coordinate plane or the core affects also represents these values of a stimulus (Figure 2). The hedonic value can induce emotional valence, while the safety value can induce emotional arousal. The safety value is related to unexpected stressful events, which evokes “fight or flight” responses, or fear and anger emotions. Fear is the emotion that is induced by the threat, while anger is the response to fight against the prediction (Figure 2; Wang and Pereira, 2016). We proposed that fear and anger are twin emotions, and two sides of the same coin. For the FND patients, they have some bad experiences which form a wrong prediction, thus an exaggerated prediction error, and exaggerated emotional reaction and behavioral changes.

Indeed, many studies have established the role of the monoamine neuromodulators in valence and arousal. Mesolimbic DA system in processing reward and pleasure (Posner et al., 2005), and 5-HT has been suggested in punishment (Dayan and Huys, 2009). DA has been proved to the substrate for reward prediction, while 5-HT has been suggested for aversion prediction (Chamberlain et al., 2006; Crockett et al., 2009). The hedonic hypothesis of DA started from Bozarth and Wise (1980), who first proposed that DA in the brain plays a critical role in subjective pleasure. The reward effects of mesolimbic system in reward have perhaps been most convincingly demonstrated in self-stimulation studies on nucleus accumbens and caudate nucleus (Mora et al., 1980). Moreover, tons of studies, notably pharmacological and behavioral studies have confirmed medial prefrontal DA system in reward behavior (Keleman et al., 2012; Liu et al., 2012). This hypothesis has significantly affected the neuroscience field about affective and drug addiction, for example, the first line treatment for affective disorders are still targeting the monoamine neurotransmitters. However, the roles of DA in the affective disorders are still the subject of research, such as the prediction error. Recently, we also suggested that the DA neuronal activity is affected by norepinephrine (NE), which is the substrate for surprise. Therefore, the prediction error is actually worked by DA together with NE. Thus the real happiness is affected by both real reward together with surprise (Happiness = Occurrence–expectancy), or (Happiness = reward–predicted reward) (Hu, 2016). Russell (2003) said: recognizing emotion is a matter of matching an acquired script with the features of a perceived event. DA may potentially participate in the onset and development of FND. DA influences multiple brain functions–including concentration, learning, memory, mood, motor control, and sleep. People who are deficient in DA lack motivation and drive, and they also become fatigued, apathetic, and possibly depressed (Voon et al., 2016).

### Basic Emotional Theory

Emotion is the least studied subject among life sciences, so far, the most advanced emotional theories are the “Basic emotional theory” and “Dimensional theory” (Lindquist and Barrett, 2012; Lindquist et al., 2013). The prediction models (actually are dimensional models, see Figure 2) we mentioned before suggested that behaviors of FND patients are due to traumatic experiences induced abnormal predictions (Hailes et al., 2019). The dimensional theory really suggests that emotions are due to two major dimensions: one is prediction (unexpected, or uncertainty induced arousal), the other is valence (Figure 2). They are due to two features of one stimulus: whether it is expected (or whether it fits to our safety needs); or whether it fits to our physiological need (Gu et al., 2016). On the contrary, the Basic emotional theory proposed that all emotions are composed of several limited basic emotions such as *joy*, *sadness*, *fear*, and *anger*; which are biologically basic and inherited (Gu et al., 2019b). Basic emotions evolved to handle fundamental life tasks, for example, fear and anger can aid survival by influencing an organism to either flee for safety or fight to defend itself. Even though emotions can be shaped by culture and learning, all humans and animals possess the capacity to experience and perceive the same basic emotions. Barrett et al. (2007) suggested that basic emotions and dimensional theory are contradicted with each other. However, in one paper, we suggested that basic emotions can be compromised with dimensional theory in that the basic emotions can also be located in the emotional dimensions (Gu et al., 2019a). The reason for them to be basic is due to the fact they are located on the poles of dimensions: the fear/anger located on the top of the arousal pole; the sadness on the negative pole of the valence dimension, and the happiness on the positive pole of the valence dimension (Figure 2).

In addition, we also suggested that the basic emotions share specific neural basis, because they can be differentiated with monoamine neuromodulators, such as dopamine (DA), serotonin (5-HT) (Dayan and Huys, 2009), or norepinephrine (NE) (Wang et al., 2020). Indeed, even though invertebrate brain structures are totally different those of vertebrate, they have similar basic emotions (Dayan and Huys, 2009; Matsumoto and Hikosaka, 2009; Fiorillo, 2013; Gu et al., 2019b). In addition, monoamines have similar functions in both vertebrate and invertebrate. Therefore, monoamine neuromodulators might be the primary substrate for basic emotions. Thus we introduced a new emotional theory based on the three monoamines, which can be called “three primary color model of basic emotions” (Wang et al., 2020). Different basic emotions, such as fear (anger), joy, disgust (sadness) consistently evoke distinct bodily sensations. For example, 6 months old children can differentiate discrete patterns of bodily sensations with joy, fear, and disgust, and the emotion related body sensation develops with child development. These developing emotion-related bodily sensations may shape the way children perceive, label, and interpret their emotions.

### Three Primary Emotions

**FIGURE 3:**
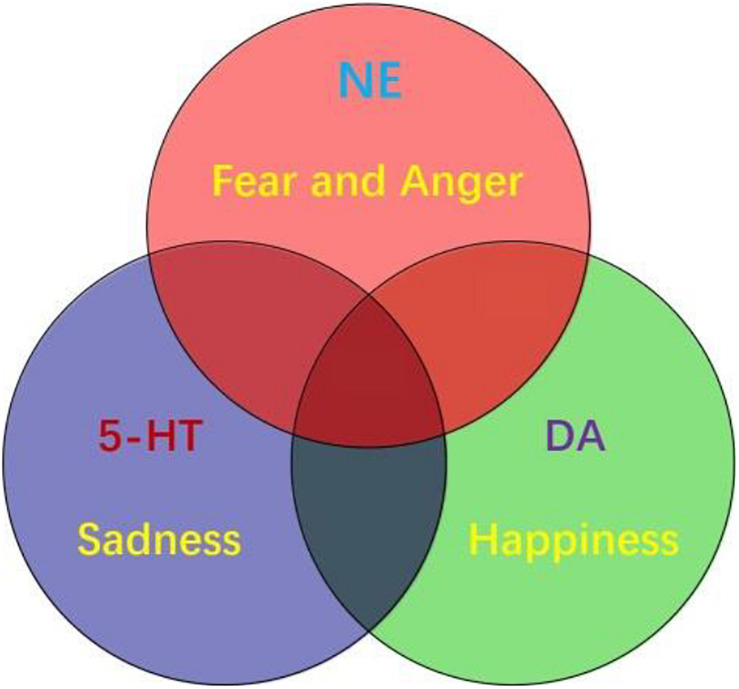
Three primary emotions, or three primary color model of basic emotion. This model proposes that the three monoamines are the substrate for three core features of all emotions: DA-reward, NE-stress, 5-HT-punishment; or primary emotions: DA-joy, 5-HT-sadness, NE-(fear and anger). Because fear and anger are one emotion, they are two-sides of one same coin. The three core emotions can induce three kinds of behaviors: stress (fight-or-flight), reward (relax and enjoy), punishment (freeze and inhibition). FND patients might have strong stressful experiences and fearful behaviors, such as frozen or tremor (seizure).

Emotion is as complicated as the colors. If not for the physiological study that there are three kinds of color cells in the retina, we will never believe that there are three kinds of primary colors. It might be also true for the emotions, the three monoamine neuromodulators are the substrates for basic emotions (Figure 3). DA gives the value of the objects: whether they fit our physiological needs (valence); while the NE gives the value of the safety (the way the objects appear, or whether they are unexpected, or arousal). Valence means how much we like the objects, arousal means if the objects are expected (prediction error) (Gu et al., 2018). Valence is dominated by DA, while arousal is affected by NE. The major function of NE is “fight-or-flight,” or “fear or anger” emotions (Wang and Anderson, 2010). Both fear and anger are evolved to cope with dangerous and unexpected situations (Anderson and Adolphs, 2014). For example, when an ox meets wolves in the wild, NE is released in both ox and wolves, but the action of the ox is flight (fear), while the behavior of the wolves is fight (anger). So fear and anger are twin emotions coming from the same neuromodulator NE (Figure 3), and both fear and anger occur at surprising ways things occur: it they occur as expected, people will not feel scared or angry.

In all, there are considerable evidences suggesting that monoamine (including DA, NE, and 5-HT) are the neural basis for emotions (Dayan and Huys, 2009; Matsumoto and Hikosaka, 2009; Fiorillo, 2013; Gu et al., 2019b). Therefore, monoamine might be the primary substrate for emotions; so we introduced the new emotional theory based on the three monoamines, which can be called “three primary color model of basic emotions” (Figure 3). In this model, we hypothesized that the substrates for these dimensions are the monoamines, or three pole of the emotional dimension: DA and 5-hydroxytryptamine (5-HT) represent two poles of the horizontal dimension, while the norepinephrine/epinephrine represents the vertical dimension (Figures 2, 3, Gu et al., 2016). Therefore, multivariate emotions are composed of three core affects, like the colors are composed of three primary colors. This might be the first theory to connect the monoamine neurotransmitters with emotional dimensions (Figure 3). Like three primary colors, there might be three primary emotions: *joy*, *sadness*, *stress* (*fear and anger*).

## Monoamine Neuromodulators in FND

The three monoamines norepinephrine (NE), dopamine (DA), and serotonin (5-HT) might be the neural basis for the three basic emotions, and represent the three poles on the emotion dimensions (Figures 2, 3). While NE, DA, and 5-HT are certainly not the only neurotransmitters involved in emotion, there is considerable evidence that these neurotransmitters play essential roles in emotions. Tons of studies from various research fields support that three monoamines, NE, DA, and 5-HT are involved in emotions (Gu et al., 2016). In addition, monoamine neuromodulator has been proved to be involved in FND symptoms. For example, the dopaminergic system plays an important role in motor control (such as DA in striatum), reward, and cognitive function. Dysfunction of dopamine in striaturn has been proved to be involved in Parkinson’s disease, which might also be the reason for tremors in FND patients. In addition, dopaminergic receptors are widely expressed in the body and function in the peripheral and central nervous system (CNS). Dopaminergic signaling pathways are essential for maintaining physiological processes, and imbalances in activity which may lead to neurological dysfunction (Klein et al., 2019). For example, one study reported that a 42-year-old female patient with conversion disorder used low-dose amisulpride to bring substantial and lasting improvement. Amisulpride, as a selective antagonist of D2 and D3 dopamine autoreceptors, can selectively block presynaptic D2 and D3 autoreceptors, resulting in increased dopaminergic transmission in the several cortical and limbic zone. It will reverse the reduction in activity of the frontal lobe and subcortical dopaminergic circuits that may be involved in the control of hysterical paralysis (Oulis et al., 2009). This suggests that increased DA can alleviate the symptoms of conversion disorders.

The major function of DA has been suggested to be reward, or reinforce natural rewarding behavior; or DA is implicated in goal-directed behaviors (Wise, 2004). DA is thought to be a signal in the brain by signaling the discrepancy between the values of predicted rewards and actual rewards (Schultz et al., 1997; Kim et al., 2015). In contrast to the suggestion that DA neurons majorly signal rewarding signals, Schultz (2015) and Ungless and Bolam (2004) argued that DA neurons largely ignore averseness, for the activities of these DA neurons were indeed activated by reward but inhibited by punishment (Matsumoto et al., 2016), and it is also found that DA release was increased by sucrose and decreased by quinine (McCutcheon et al., 2012), suggesting that DA activity is inhibited by aversive stimuli (Fiorillo, 2013). However, there are some studies suggesting that DA might be involved in aversive stimuli (such as foot shocks, hind paw injection, or social defeat). This might be due to the fact that midbrain DA systems are composed of heterogeneous DA subgroups of neurons (Lammel et al., 2014). However, Schultz (2015) argued that the excitation of DA neurons caused by aversive stimuli may be due to a “generalization” or “spill-over” of rewarding stimuli. In one influential paper, Fiorillo (2013) made a conclusion that DA neurons only represent prediction errors about reward instead of averseness. The experiments were designed to show that the activities of DA neurons were similar to aversive stimuli to neutral stimuli; the activities of most the DA neurons were not affected by prediction to aversive events like to reward events.

The synthesis of NE is mainly carried out in the adrenal medullary chromaffin cells (AMCCs). Tyrosine in the blood is taken up by the cytoplasm of adrenergic nerve endings, and converted into DA under the catalysis of tyrosine hydroxylase and decarboxylase, and then catalyzed by dopamine β-hydroxylase to synthesize NE, which is stored in the capsule bubble. When the nerve impulse reaches the nerve terminal, the vesicle approaches the presynaptic membrane and releases NE into the synaptic cleft in the form of cleavage and efflux, which stimulates the corresponding receptors on the post-synaptic membrane to produce a series of physiological effects (Cho et al., 2002). The researchers summarizing the findings about the anatomy and physiology of the noradrenergic system in the CNS, the importance of NE for maintaining cognitive processes such as perception, attention, especially memory consolidation and recovery. In the peripheral nervous system (PNS), NE is released from the adrenal gland as a kind of hormone, to directly increases heart rate, triggers the release of glucose from energy stores, and increases blood flow to skeletal muscle, and induce fight-or-flight response (Prokopová, 2010). In the CNS, NE is released from the locus coeruleus (LC), which is the principal site for brain synthesis of NE. The LC is the largest of the noradrenergic groups and provides the principal source of NE innervation to the entire cerebral cortex as well as the hippocampus, amygdala, cerebellar cortex, and spinal cord. LC-NE system has been suggested to be implicated in higher cognitive processes such as attention, memory, perception, emotion, and motivation (Berridge and Waterhouse, 2003). Dysregulation of LC-NE neurotransmission may contribute to cognitive and/or arousal dysfunction associated with a variety of psychiatric disorders, including attention-deficit hyperactivity disorder, sleep and arousal disorders, as well as certain affective disorders, including post-traumatic stress disorder (Aston-Jones and Cohen, 2005).

The LC-NE system can activate amygdala, which responds by sending signals to the hypothalamus, which in turn stimulates the PNS NE activity. “Core” FND symptoms include weakness, seizures or paralysis, other movement disorders (such as tremor, dystonia, abnormal gait, and myoclonus), and sensory disorders (including vision, physical sensation, and auditory impairment) (Jones et al., 2016), as well as symptoms including pain, fatigue, sleep disturbance, inattention, and memory cognitive impairment (Glass et al., 2018; Nicholson et al., 2020). It is not difficult to find that most of the core symptoms of FND and other related physical and psychological symptoms are consistent with the deficits of NE, because NE has been known to have the function of arousal, and antiepileptic effects. We can make a bold speculation that the abnormal performance of FND may be related to NE deficit.

The neuromodulator serotonin (5-HT) has been implicated in a large number of affective and executive functions, such as depression or sleep. Recently, more and more work highlighted its role in aversive processing (Crockett et al., 2010), another major function is behavioral inhibition (Crockett et al., 2009). Crockett suggested that “Few would disagree that serotonin is involved in aversive processing.” (Crockett et al., 2009). Many studies have confirmed its role in encoding aversion, for example, aversive events activate 5-HT releasing, and depleted 5-HT reduced behavioral suppression. However, some other evidence points to the other direction: selective 5-HT reuptake inhibitor is a major therapy for depression. The major reason is that the major function of 5-HT is predicting aversive outcome, or inhibit aversive reaction (Chamberlain et al., 2006; Crockett et al., 2009).

**FIGURE 4:**
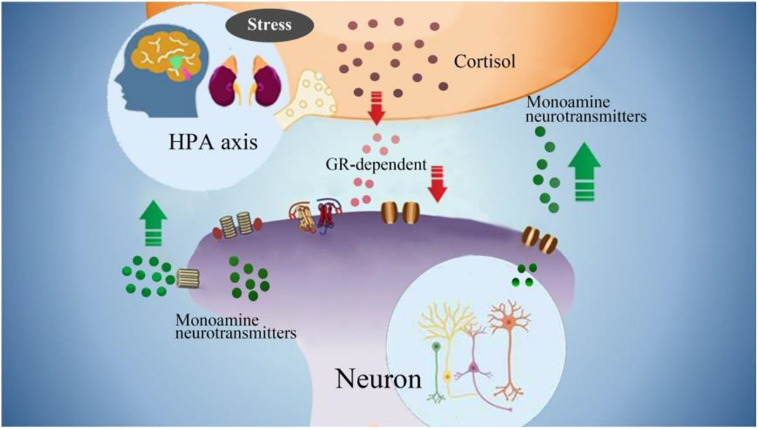
The interactions between monoamine neurotransmitters with HPA axis. Stress induced emotional arousal and monoamine neurotransmitters can enhance HPA activity, which leads to excessive secretion of cortisol. On the other hand, the excessive cortisol, in turn can induce dysfunctional release of monoamine neurotransmitters (increase/decrease), which may be the pathogenesis of FND.

Neurons containing 5-HT in the midbrain directly innervate the corticotropin releasing hormone (CRH) cells located in the paraventricular nucleus of the hypothalamus. The serotonergic input into the paraventricular nucleus mediates the release of CRH, leading to the release of adrenal cortex hormones, thereby triggering the secretion of adrenal cortex glucocorticoids. 5-HT1A and 5-HT2A receptors are the main receptors that mediate serotonergic stimulation of the hypothalamic-pituitary-adrenal (HPA) axis (Hanley and Van de Kar, 2003). This connection seems to depend on corticosteroid levels. Glucocorticoid receptor imbalance can change the negative feedback regulation in the HPA axis (Figure 4). Serotonin transporter (5-HTT) regulates serotonin transmission by removing 5-HT from the synaptic cleft, which leads to serotonergic dysfunction (Cubała and Landowski, 2006; Phi Van et al., 2018). Based on the above evidence, some clinical patients may benefit from selective serotonin reuptake inhibitors (SSRIs), whose major function is increase the concentration of NE and 5-HT. Indeed, the frequency of psychogenic non-epileptic seizures can be reduced by using SSRIs treatment (Haykal and Smith, 2015). In addition, these monoamine neurotransmitters can affect each other, for example, DA neurons have two 5-HT receptors, 5-HT1A and 5-HT2A. 5-HT1A can promote the release of DA, and 5-HT2A can inhibit the release of DA (which is the opposite of the regulation mechanism of 5-HT on the release of glutamate from cortical glutamatergic neurons). NE neurons have two-way feedback regulation on the release of 5-HT. We have understood the possible connection between 5-HT and FND, and then we will discuss the changes in NE and DA in FND patients.

## Emotional Behaviors in FND

Emotion is a kind of internal drive, which can be expressed as external behaviors. Emotion induced external behaviors are genetically hardwired and are crucial for animal’s survival. Several major categories of behaviors are developed, such as feeding, reproduction, aggression and sleep, which are observed across animal species. These instinct behaviors are robust, fluent and adaptive to an animal’s internal and external context (Kim et al., 2017). Most of the FND symptoms, such as limb weakness or seizures, are derived from these emotional behaviors.

### Stress

Stress is closely associated with most mental disorders, either through early life trauma as a presumed “vulnerability factor” or through late life events prior to symptom onset as a “precipitating factor” (Duffy et al., 2018). Many scholars attribute FND to psychological stressors, especially historical trauma. It is traditionally believed that psychosocial stressors can be identified around the onset of symptoms. Recent research has shown that compared with depression and healthy controls, FND patients have much more serious stressful life events, especially in the month before the onset of symptoms, about 56% of FND patients have at least one serious stress event (Nicholson et al., 2016). Childhood trauma is a risk factor for the development of FND. A recent meta-analysis of a case-control study found that the incidence of stressors in childhood and adulthood was higher in FND patients than in controls (Bailey et al., 2018). Studies have also shown that abnormalities of the left anterior insular nerve caused by childhood abuse may be the basis of the pathophysiology of FND. Female patients with FND showed a significant association between functional neurological symptoms and decreased gray matter volume of the left anterior insula (Perez et al., 2017).

### Arousal

The relationship between arousal or stress and functional symptoms has been examined to investigate physiological measures of arousal. It is showed that patients with mixed active FND symptoms compared with those with anxiety disorders or healthy volunteers had greater baseline arousal levels, as measured by spontaneous fluctuation in skin resistance along with failure to habituate skin conductance to repeated auditory stimuli (Voon et al., 2016). FND patients might have sensitive safety needs due to early stressful life events, so they might easily feel the stresses in adult lives. FND patients might have strong stressful experiences and fearful behaviors, such as frozen or tremor (seizure). In the periphery nervous system, NE works to increase heart rate and blood flow to skeletal muscles. In the CNS, NE works to induce wakefulness and emotion arousal. NE is released from the locus coeruleus (LC) to keep the brain alert to unexpected stimuli (or prediction error). LC sends projections to many brain areas, such as amygdala, which might be the locus for fear memory. Bakvis et al. (2010) further showed that patients with seizure have increased basal diurnal cortisol levels associated with a history of sexual trauma and lower heart rate variability at baseline, suggesting greater sympathetic activity.

### Fight or Flight

The fight-flight-freeze response is a natural reaction to danger, and it is a type of response that helps the individual reacts to perceived threats, like an oncoming car or growling dog. Specifically, fight-or-flight is an active defense response for the individual to fight or flee. The heart rate gets faster, which increases oxygen flow to major muscles, and the pain perception drops, and hearing sharpens. These changes help the individual to act appropriately and rapidly. Freezing is fight-or-flight on hold, where the individual further prepares to protect himself. It’s also called reactive immobility or attentive immobility, which involves similar physiological changes, but instead, the individual stays completely still and get ready for the next move. The fight-flight-freeze response is a natural reaction to threat, and it is a kind of behavior that helps the individual to react to perceived threats. The term “fight or flight” (also known as acute stress response) are developed to react to the changes that occur in response to perceived threat for our ancient ancestors. It is first described by an American physiologist Walter Bradford Cannon (Zheng et al., 2016). The theory first stated that all animals react to threats with an arousal of sympathetic nervous system to prepare the animal for fighting or fleeing. The sympathetic nervous system then stimulates the adrenal glands, triggering the release of catecholamines (including adrenaline and noradrenaline), which then induce a chain of reactions, such as increased heart rate, blood pressure.

### Freeze

In addition to “fight or flight,” freezing is a kind of reactive immobility or attentive immobility, which involves similar increase in heat rate and blood flow, but body stays completely still. This is an automatic reaction, which might work through amygdala, and the amygdala responds by sending signals to hypothalamus, and the hypothalamus works by stimulating sympathetic nervous system (Diez et al., 2020). When the body is faced with perceived threat, the body would automatically reacts with fight-flight-freeze response to keep the individual safe. It is very normal in the wild animal world, when a prey meets a predator, for instance, a rat meets a cat. You can see that, all of a sudden, the rat seems to sense the deadly predator is near and freezes in place. You can see that its ears and nostrils are twitching, and its eyes are darting back and forth, but no movement is apparent. Evolutionarily, freezing is a more advanced response to sense the real threat than running around. Many studies have done about freezing in rats in lab, for example Ledoux and Brown (2017) found that the neural pathway is from auditory thalamus to amygdala.

Severe fear can cause freeze behavior that happens normally in both healthy people and FND patients. Indeed, behavioral inhibition is a personality type, like shyness, that shows a tendency toward nervousness in new environment. Similarly, FND patients usually show body trembling, shaking, tremors and vibrating and also anxiety disorders, including generalized anxiety, social anxiety, panic disorders etc. Many healthy people experienced body tremors when they are stressed. Tremors activated by the stresses cause body-wide adrenaline changes to boost the energy in the body, and prepare the body for fight or flight, for which freeze is prepared. Tremor induced by tightening the muscle to be more resilient to damage, stimulating the nervous system to run faster, or to prepare for fight or flight. All the changes that cause the body to tremble is an “emergency boost of resources” to deal with the threat (fight-flight-freeze). When stresses are too frequent, the body becomes hyper-stimulated, and tremor can occur without reason or involuntarily. Trembling, and shaking symptoms are the most common types of symptom in FND patients. Tremors can develop into spasms, jerking movements, even looking like Parkinson’s disease, or seizures (which are called non-epileptic seizures, dissociative seizure, or psychogenic seizures.). Sometimes, the response is overactive, which is common in people who have experienced traumatic stress before. In this case, the brain reacts to related events with overactive response.

### Inhibition

Many studies have focused on the role of behavioral inhibition in sadness and depression (Gladstone and Parker, 2006). Our understanding of “behavioral inhibition to the unfamiliar” has grown tremendously over the past 30 years. Behavior inhibition is defined as the persistent tendency to show extreme avoidance, reticence in novel environment or with unfamiliar people. Behavioral inhibition has been shown to be a risk factor for social anxiety disorders and depression (Hirshfeld-Becker et al., 2008). Similar studies have also found that the behavior inhibition by a postpartum depressive mom can induce development disorders in child (Oyetunji and Chandra, 2020). Similarly, a series of studies have focused on the question of an “inhibition” of motor execution in FND patients, and found that preparation to move is intact but that execution is inhibited by prefrontal regions (Halligan et al., 2000).

Serotonin (5-HT) in the CNS is thought to be the substrate of behavioral inhibition. Indeed, 5-HT has been shown to induce inhibition of locomotion, stimulation of egg laying, and pharyngeal pumping in *Caenorhabditis elegance* (Dayan and Huys, 2009). Similar studies also showed that the neural activity of the 5-HT interneuron increases when the *C. elegance* stops moving forward (Mori et al., 2019); another study found that 5-HT can cause food-deprived animals to run slowly at a field with its food bacteria (Sawin et al., 2000). The forebrain 5-HT system is a crucial for the impulsive behaviors, and low level 5-HT in this area promotes impulsive behaviors, such as motor impulsivity, which means the failure to suppress inappropriate actions (Miyazaki et al., 2012). Consistently, another study found that that immaturity of 5-HT-mediated behavioral inhibition contributes to the adolescent behavioral impairment.

### Weakness

Acute stress can induce fight-or-flight, while long-term chronic stress might induce tiredness and weakness, which might be due to decreased activity of NE and increased release of 5-HT. Mechanisms of weakness in FND patient might also be similar to those involved in hypnotic suggestion. Hypnosis is an alteration in consciousness with heightened suggestibility and decreased awareness, and it is well known that the major function of NE is waking (Bacon et al., 2020), while the function of 5-HT is sleep (Seifinejad et al., 2020; Valentino and Volkow, 2020). Oakley (1999) defines hypnosis as the “withholding of representations from entry into self-awareness…as a result of the inhibition by the central executive system with a separation of the executive system and awareness.” Similarities exist on a phenomenological level between hypnosis and functional symptoms (e.g., in the dissociation between subjective intention to move and the actual movement). Subjects with high susceptibility for FND are also more likely to be hypnotizable (Voon et al., 2016).

### Chronic Pain

Chronic pain can happen in FND patients, especially women, and it is characterized by widespread pain, accompanied with sleep problems, profound fatigue, and fatigue syndrome. Sometimes they also accompany with other symptoms, such as morning stiffness, headaches, and tingling and numbness in limbs, even irritable belly. These symptoms will not recover with rest, instead they are due to emotional trauma.

## Neural Modulator Changes Involved in FND

Functional neurologic disorder Symptoms occur as a result of problems in the brain, which fails to send and/or receive messages correctly, and result in a variety of sensory and movement disturbances. In a lot of cases symptoms can be extremely disabling, but the brain problems are only functional for emotions. The neural basis that works for emotions has been intensively studied in recent years. And most of the studies suggest that specific basic emotion can be located in one specific brain nucleus (Lindquist et al., 2013). Indeed, many neuroimaging studies found some evidence for basic emotions (such as amygdala for fear, insula for disgust, anterior cingulate cortex for sadness, and orbitofrontal cortex for anger). However, most of the work focused on the neural network for emotions, such as the Papez’s circle, suggested that the limbic system is the neural basis for emotions. For example, the brainstem is home for a group of modulatory neurotransmitter pathways, such as locus coeruleus (NE) and ventral tegmental area (DA), and raphe nuclei (5-HT). However, recent studies with neuroimaging met some problems, for it is hard to differentiate the basic emotions with different brain structures. Actually the emotions are often wildly spread and involve the whole brain to work together, instead of only specific brain structures. Hence, we introduced an alternative approach, the neuromodulators (Gu et al., 2016), and we suggested that the emotions are majorly due to neuromodulators. Indeed, FND patients have been found some dysfunctions of monoamine neuromodulators in the brain, which are responsible for emotion regulation, execution of control processes and movement, such as the prefrontal cortex, inferior frontal gyrus, insula, and parietal cortex (Table 1). Especially the LC-NE system and amygdala, the part of brain that is linked to negative emotions, is found to be enlarged and activated during stressful events.

**TABLE 1:** Emotional processing in FND.

Abnormal symptoms	Emotional processing	Activation brain areas	References
Stress/tremor Involuntary movement	Stressful/aggressive	Amygdala/LC-NE increased	Szaflarski et al., 2018
Myoclonus/seizure Jerk/twitch	Nervous/Fight-or-flight	Periaqueductal gray LC-NE increased	Aybek et al., 2015
Sensory changes Dry mouth Irritable bowel Urinary urgency	Nervous/Interoception	Left Heschl’s gyrus	Espay et al., 2018c
Sleep disturbance Insomnia/hypersomnia	Aversive prediction error/Calm	Periaqueductal gray 5-HT changed	Aybek et al., 2015
Pain, dystonia headache tingling and numbness of arms and legs Cognitive changes Brain foggy Forgetfulness	Enhanced Somatic activity No motivation	Precentral Prefrontal Striatum DA activity decreased	[Bibr B35][Bibr B100][Bibr B37][Bibr B16][Bibr B34]
Poor concentration Depression Tiredness Dysphonia Stuttering	Nervous/Freezing	LC-NE/HPA axis decreased	Sojka et al., 2019
Limb weakness Paralysis Dropping things Uneven steps/drag leg knee buckling	Nervous/Freezing	LC-NE/HPA axis Decreased	[Bibr B7][Bibr B79][Bibr B7][Bibr B79]

In addition to the monoamine neuromodulators, other hormones and neurotransmitters may also be involved in a secondary way. For example, corticotropin-releasing hormone, which was named the stress hormone, might be a pathway for central norepinephrine (NE). The response to stress is mediated by two main pathways: the autonomic sympathetic response with rapid adrenaline/norepinephrine secretion and the slower response of the HPA axis with cortisol secretion. The relationship between stress, HPA axis and clinical pathology is highly complicated. In an experiment of 18 FND seizure patients and 19 healthy controls, the basic diurnal cortisol levels of seizure patients increased significantly (Bakvis et al., 2010). Although historically coexisting psychological stressors have been used as supporting factors for FND diagnosis, many patients with functional neurological diseases deny the existence of these stressors. For example, a study conducted in 2015 sampled the blood of 33 FND patients and 33 gender-matched controls and found no significant difference in circulating cortisol (Maurer et al., 2015). Therefore, there is controversy about the concept of stress level and HPA axis activation in the pathology of FND. A recent study conducted a Trier social stress test on 16 FND (DSM-5 standard) patients and 15 healthy controls. Salivary cortisol was used to assess the HPA axis response, and salivary α-amylase was used to assess autonomous sympathetic response. The results showed that the two stress indicators of FND patients were significantly higher than those of the control group, which confirmed that HPA axis sympathetic hyperactivity is related to FND caused by life adversity (Apazoglou et al., 2017, 2018). This further confuses HPA axis involvement and whether stress is a factor.

Excessive secretion of cortisol in response to stress can cause hippocampal damage and disrupt negative feedback, leading to uncontrolled secretion and further damage. Hypercortisolemia and changes in synaptic plasticity, decreased neurogenesis, neuronal atrophy, trigger hippocampal changes, which can lead to nerve cell death (Kumar et al., 2019). The HPA axis participates in the steady-state response to environmental changes. Hyperfunction of the HPA axis may be the pathogenesis of FND. Due to the destructive effects of overactive HPA axis on brain tissue, 5-HT levels will decrease (Ambrus et al., 2016). Therefore, it may provide us with a direction to explore the potential mechanisms and biomarkers of the true diagnosis of FND (Figure 4).

## Conclusion and Discussion

Functional neurologic disorders are neurologic malfunctions which are induced by psychological distress, and are expressed in the form of physical symptoms. These symptoms, however, have no underlying physical cause and are often associated with an emotional or psychological crisis. In the original formulation of hysterical conversion by psychodynamic theories, emotions play a crucial role in the etiology of FND. It is suggested that FND is the body expressing the unprocessed stress in an extreme way. However, recent studies suggested a multi-model for its etiology, for example, there is increasing evidence that patients with FND exhibit activation of the monoamine neuromodulators. We believe that stress changes can directly cause FND symptoms through the monoamine neurotransmitters (Kortekaas et al., 2005; Beaulieu and Gainetdinov, 2011). For chronic neurological diseases such as FND, the influence of monoamine neurotransmitters is particularly important. FND has always been suggested to be a “contradictory” disease, for example, Janet (1907) proposed that the monoamine dysfunction may be universally defective, and emphasized the role of abnormal concentration of monoamine in FND. Whitlock (1967) and Ludwig (1972) agree with Janet. Paradoxically, FND is related to “selective depression of awareness of a bodily function,” and such patients have characteristic “subtle disturbance in cognitive functioning” (Stone, 2020). Now, diagnosis can be made in an inclusive manner by identifying the neurological symptoms specific to FND, without relying on whether there is psychological pressure or implied historical clues (Espay et al., 2018a). In addition, although historical trauma and stress predisposing factors are not invariable, they are very common in FND. Tests and treatments need to be comprehensively evaluated, and the impact of disease beliefs and disease patterns on society is still very important (Keynejad et al., 2017).

Functional neurologic disorder is a neglected but possibly reversible source of disability. Monoamine neuromodulation products and HPA axis hyperactivity are possible pathological changes of FND. The limitations of this article include the moderate amount of literature. There is controversy about how to conceptualize FND, including whether the disease is classified as stress-related, and whether emotional stress directly affects monoamine neuromodulation and causes FND. First, multiple core symptoms and related symptoms mean that a single core FND symptom should not be assumed to be the most significant aspect of the disease. Therefore, other simultaneous neuromodulation changes are also important to explore the mechanism of FND symptoms. FND is a neuropsychiatric disease with highly variable semiotic manifestations, multiple comorbidities and related social and psychological factors. A single simple mechanism index may not be the best choice. Is the HPA axis hyperfunction and monoamine neuromodulation turbulence summarized as the possible pathogenesis of FND obsolete, or is there new hope? In any case, it is time for psychiatry to stop consciously or unconsciously suppressing this important underlying disease.

## Author Contributions

QX and FW wrote the first draft. MJ, FL, RF, SJ, BY, and TW made major revisions of this article. SX, FW, and JH provided the critical revisions. All authors approved the final version of the manuscript for submission.

## Conflict of Interest

The authors declare that the research was conducted in the absence of any commercial or financial relationships that could be construed as a potential conflict of interest.
